# Prognostic Models Using Machine Learning Algorithms and Treatment Outcomes of Papillary Thyroid Carcinoma Variants

**DOI:** 10.1002/cnr2.70071

**Published:** 2024-11-29

**Authors:** Sakhr Alshwayyat, Haya Kamal, Owais Ghammaz, Tala Abdulsalam Alshwayyat, Mustafa Alshwayyat, Ramez M. Odat, Hamdah Hanifa, Wafa Asha, Nesreen A. Saadeh

**Affiliations:** ^1^ Research Associate King Hussein Cancer Center Amman Jordan; ^2^ Internship Princess Basma Teaching Hospital Irbid Jordan; ^3^ Applied Science Research Center Applied Science Private University Amman Jordan; ^4^ Faculty of Medicine Jordan University of Science & Technology Irbid Jordan; ^5^ Faculty of Medicine University of Kalamoon Al‐Nabk Syria; ^6^ Department of Radiation Oncology King Hussein Cancer Center Amman Jordan; ^7^ Internal Medicine Department Jordan University of Science and Technology Irbid Jordan

**Keywords:** clinical decision‐making, machine learning, papillary thyroid cancer, prognosis, survival analysis, treatment outcome

## Abstract

**Background:**

Hürthle cell (HCC) and columnar cell variants (CCV) are rare subtypes of thyroid cancer.

**Aims:**

This study used machine learning (ML) to evaluate treatment effectiveness and develop prognostic models.

**Methods:**

Chi‐square tests, Kaplan–Meier curves, log‐rank tests, and Cox regression were used. Five ML algorithms constructed prognostic models predicting 5‐year survival, validated using the AUC of the ROC curve.

**Results:**

Among 3690 patients, 3180 had CCV and 510 had HCC. ML models showed metastasis, surgery + RT, and age were significant factors for HCC, while the N component of TNM, metastasis, and tumor size were significant for CCV.

**Conclusion:**

This study offers a comprehensive approach for treating and assessing prognosis in PTC variants. The ML models developed offer practical tools for personalized clinical decision‐making.

## 1 Introduction

Papillary thyroid carcinoma (PTC) is the most prevalent form of endocrine malignancy, and its incidence is increasing worldwide. Generally, it has a favorable prognosis, with a 10‐year survival rate > 95% [1, 2]. However, certain subtypes of PTC may be more aggressive than typical PTCs [3, 4], which can lead to poorer survival rates and higher rates of metastasis and recurrence [5]. PTC variants include Hürthle cell (HCC) and columnar cell variants (CCV). HCC, also known as oxyphilic thyroid carcinoma represents a rare and aggressive form of PTC, constituting about 3%–4% of all thyroid cancer cases [6]. It makes up about 1%–11% of PTC cases and is characterized by an abundance of oxyphilic granular cytoplasm due to the formation of numerous unusual mitochondria [7, 8, 9]. CCV, which accounts for 0.15% to 0.4% of all reported PTC cases [4, 10], affects a broad age range, spreads widely, and exhibits high rates of recurrence [3, 5]. This subtype is identified by the presence of nuclear stratification that is clearly visible on papillae or gland‐like structures lined with columnar cells [1, 4].

Treatment strategies for PTC usually involve a combination of surgery, radioactive iodine (RAI) therapy, and thyrotropin suppression therapy [2, 11]. Total thyroidectomy is the primary surgical approach used to manage these cases [11, 12]. Given the aggressive nature of rare PTC variants, more aggressive treatment strategies may be warranted. The effectiveness of RAI therapy remains debatable; some recent studies suggest it might improve overall survival (OS) rates, whereas others question its utility [5, 9, 13]. Building on the context of treatment strategies for PTC, artificial intelligence, particularly ML, plays a pivotal role in enhancing medical outcomes. ML, which emphasizes the refinement of learning algorithms through data analysis, has shown remarkable success in solving complex challenges in the medical field, including medical image recognition, treatment optimization, and biomedical research [14, 15, 16].

The primary objective of this study was to evaluate the efficacy of different treatment modalities for patients with PTC variants. Furthermore, we developed an ML‐based classification system designed to provide accurate and reliable predictions, thereby aiming to improve personalized care and optimize patient management. This integration of ML into treatment evaluation and prognosis underscores a significant stride toward more tailored and effective therapeutic strategies for PTC.

## 2 Methods and Materials

### 2.1 Data Extraction and Variables

Data for this study were obtained from the SEER database (https://seer.cancer.gov/), representing about 27.8% of the U.S. population, using SEER*Stat software (version 8.4.2). We used histological codes 8342 (Papillary carcinoma, oxyphilic cell) and 8344 (Papillary carcinoma, columnar cell) for HCC and CCV, respectively. Exclusions were made for cases without histological confirmation, secondary tumors, or missing data, resulting in 3690 eligible patients.

We analyzed the SEER data on patient demographics from 2000 to 2020, tumor characteristics (size, stage, and TNM classification), treatment methods (surgery, CTX, and RT), and survival outcomes. Age was divided into < 45 and ≥ 45 years; tumor size into < 2 cm and ≥ 2 cm; and staging into localized, regional, and distant. The main survival outcomes were OS, calculated from diagnosis to any cause of death, and cancer‐specific survival (CSS), calculated from diagnosis to death due to PTC variants.

## 3 Statistical Analysis

### 3.1 Data Processing and Statistical Methods

All data were analyzed using R software (v4.0.0) and the following packages: “readxl,” “tidyverse,” “Hmisc,” “data.table,” “table1,” “MatchIt,” “survminer,” “survival,” and “broom.” Chi‐square analysis was used to assess categorical variables and evaluate the clinicopathological characteristics of patients with PTC variants. The Kaplan–Mier (K‐M) method was used to estimate survival rates, and the log‐rank test was used to analyze differences between survival curves. Multivariate Cox proportional hazards models identified independent predictors of survival. A two‐tailed *p* value less than 0.05 was considered statistically significant. Five ML ensembles were employed: random forest classifier (RFC), Gradient Boosting classifier (XGBoost), Logistic Regression (LR), K‐Nearest Neighbors (KNN), and multilayer perceptron (MLP). These algorithms were selected because of their ability to handle complex multidimensional datasets.

### 3.2 Model Training

To ensure model robustness, standard scaling was applied uniformly across all features using the StandardScaler module from Scikit‐learn. This normalized the data, thereby alleviating the potential impacts of different scales among the features. Subsequently, ML models were trained on a dataset with a binary classification output predicting the target “5‐year survival.” The features included demographics, tumor characteristics, and management approaches. The dataset was randomly split into 8:2 for training and testing sets.

### 3.3 Feature Importance and Model Evaluation

The feature contribution in predicting “5‐year survival” was calculated using the permutation importance method. ROC curves and AUC scores were used to evaluate the model's discriminatory power. The roc_curve function from the sklearn. Metrics module computes false‐positive rate (FPR) and true‐positive rate (TPR). The predicted probabilities of the positive class were obtained using the predict‐proba method for each model. AUC scores were calculated using the roc_auc_score function. A custom plotting function, plot_roc_curve, and visualized ROC curves of the multiple models. Additional model evaluation included a Mean Bootstrap Estimate with a 95% confidence interval, 10‐fold cross‐validation, and a classification report for precision, recall, and F1‐score. All ML implementations were processed using the scikit‐learn 0.18 package in Python.

## 4 Results

### 4.1 Clinicopathological Characteristics of PTC Variants Patients

The study population comprised 3690 patients diagnosed between 2000 and 2020. Among them, 3180 patients had CCV and 510 had HCC. Most patients (62.8%) were 45 years or older, with a median age of 52 years. A total of 56.9% of the patients had a tumor size greater than 2 cm, with a median tumor size of 3.1 cm. The largest racial group was white, comprising 83.8% of the cases, and 11.8% of the cases were Asian. Furthermore, most cases were regional (53.4%, *n* = 1969), followed by localized (38.3%, *n* = 1413). The demographic and clinical characteristics of PTC variant patients with different treatment patterns are shown in Table 1.

**TABLE 1:** Clinicopathological characteristics.

Category characteristics	Papillary carcinoma, columnar cell (*N* = 3180)	Papillary carcinoma, oxyphilic cell (*N* = 510)	Overall (*N* = 3690)	*p*
Sex				
Female	2315 (72.8%)	403 (79.0%)	2718 (73.7%)	0.012
Male	865 (27.2%)	107 (21.0%)	972 (26.3%)	
Age				
< 45 years	1184 (37.2%)	190 (37.3%)	1374 (37.2%)	1
≥ 45 years	1996 (62.8%)	320 (62.7%)	2316 (62.8%)	
Race				
Asian or Pacific Islander	381 (12.0%)	54 (10.6%)	435 (11.8%)	0.882
Black	141 (4.4%)	20 (3.9%)	161 (4.4%)	
White	2658 (83.6%)	436 (85.5%)	3094 (83.8%)	
Tumor size				
< 2 cm	1319 (41.5%)	272 (53.3%)	1591 (43.1%)	< 0.001
≥ 2 cm	1861 (58.5%)	238 (46.7%)	2099 (56.9%)	
Stage				
Distant	296 (9.3%)	12 (2.4%)	308 (8.3%)	< 0.001
Localized	1083 (34.1%)	330 (64.7%)	1413 (38.3%)	
Regional	1801 (56.6%)	168 (32.9%)	1969 (53.4%)	
Metastasis				
No	2884 (90.7%)	330 (64.7%)	3214 (87.1%)	< 0.001
Yes	296 (9.3%)	180 (35.3%)	476 (12.9%)	
AJCC				
I	945 (29.7%)	195 (38.2%)	1140 (30.9%)	< 0.001
II	181 (5.7%)	43 (8.4%)	224 (6.1%)	
III	467 (14.7%)	53 (10.4%)	520 (14.1%)	
IV	419 (13.2%)	22 (4.3%)	441 (12.0%)	
Unknown stage	1168 (36.7%)	197 (38.6%)	1365 (37.0%)	
T				
T1	752 (23.6%)	191 (37.5%)	943 (25.6%)	< 0.001
T2	358 (11.3%)	134 (26.3%)	492 (13.3%)	
T3	951 (29.9%)	86 (16.9%)	1037 (28.1%)	
T4	316 (9.9%)	0 (0%)	316 (8.6%)	
Unknown stage	803 (25.3%)	99 (19.4%)	902 (24.4%)	
N				
N0	1204 (37.9%)	288 (56.5%)	1492 (40.4%)	< 0.001
N1	1159 (36.4%)	102 (20.0%)	1261 (34.2%)	
Unknown stage	817 (25.7%)	120 (23.5%)	937 (25.4%)	
Treatment				
Surgery	1001 (31.5%)	237 (46.5%)	1238 (33.6%)	< 0.001
Surgery + RT	2128 (66.9%)	273 (53.5%)	2401 (65.1%)	
Surgery + RT + CTX	51 (1.6%)	0 (0%)	51 (1.4%)	
Type of surgery				
Lobectomy	319 (10.0%)	84 (16.5%)	403 (10.9%)	< 0.001
Total thyroidectomy	2861 (90.0%)	426 (83.5%)	3287 (89.1%)	

### 4.2 Survival Analysis

**FIGURE 1:**
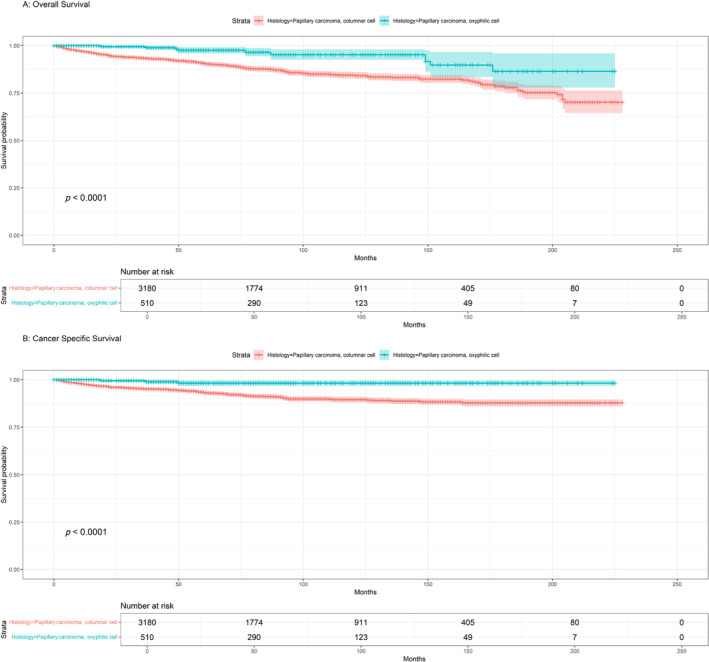
(A) Kaplan–Meier overall survival (OS) based on histology; (B) Kaplan–Meier cancer‐specific survival (CSS) for histology.

**FIGURE 2:**
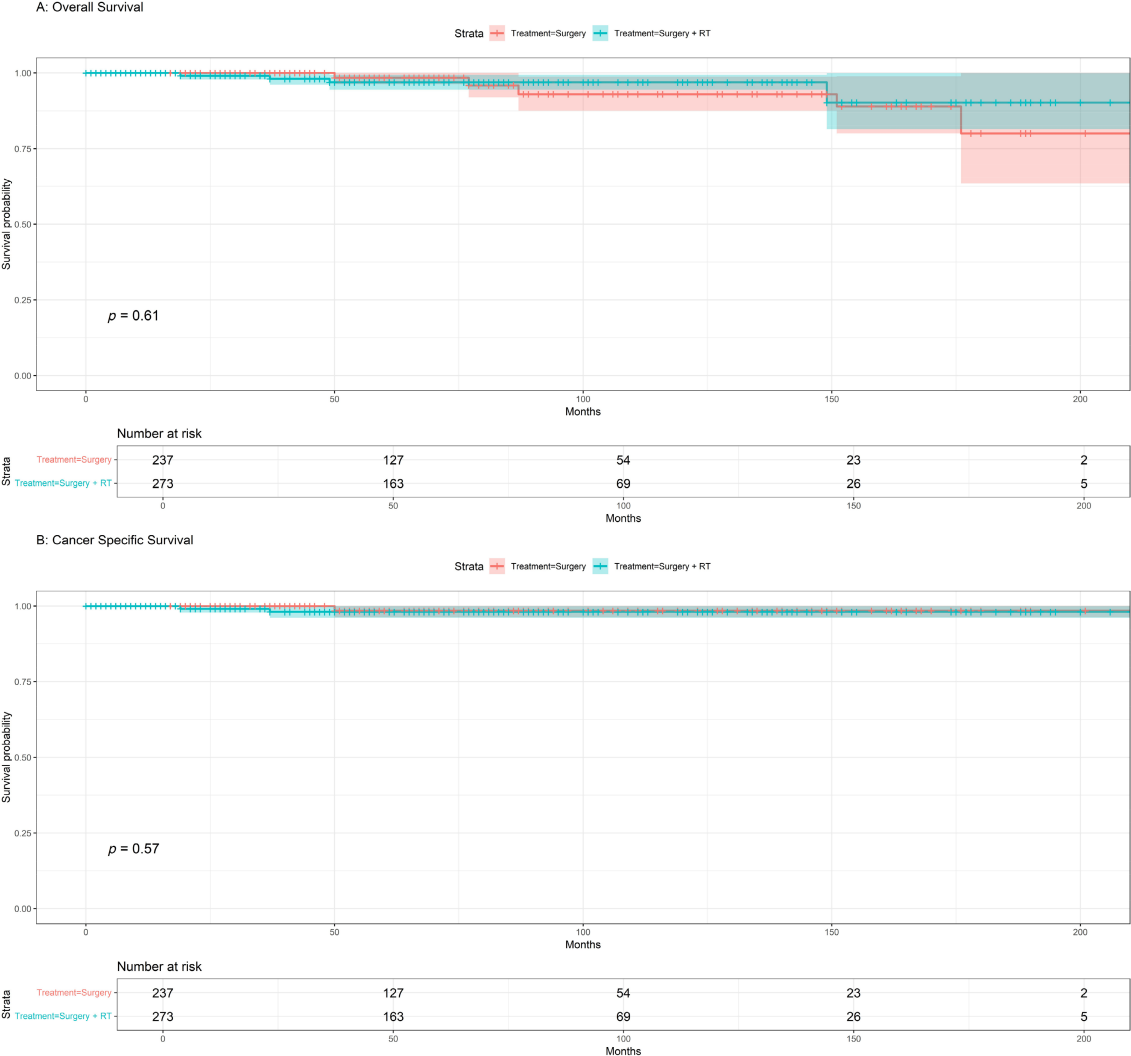
HCC: (A) Kaplan–Meier overall survival (OS) for treatment; (B) Kaplan–Meier cancer‐specific survival (CSS) for treatment.

**FIGURE 3:**
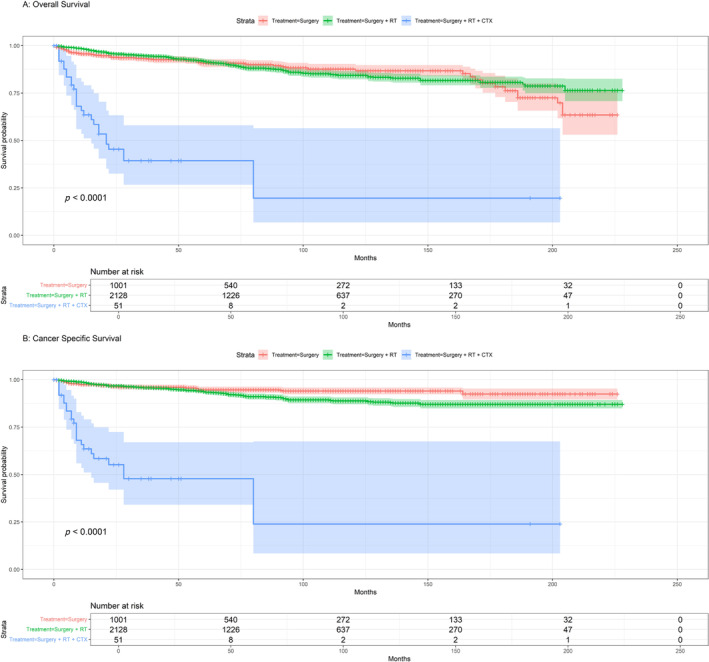
CCV: (A) Kaplan–Meier overall survival (OS) for treatment; (B) Kaplan–Meier cancer‐specific survival (CSS) for treatment.

The 5‐year OS rates of patients with CCV and HCC were 90.6% and 97.5%, respectively, and those for CSS were 97.5% and 90.6%, respectively (Figure 1). Regarding HCC, the 5‐year survival rates were remarkably high and did not show any significant differences, with OS of 98.4% for surgery alone and 96.9% for the combination of surgery and RT, and CSS of 98.4% surgery and 98% for surgery and RT (Figure 2). The 5‐year OS rates for CCV treatment options were significant, with surgery alone showing a rate of 90.7%, surgery combined with RT at 91.8%, and surgery + RT + CTX resulted in a survival rate of 39.3%. In terms of CSS, 94.7% for surgery, surgery combined with RT at 93.7%, and surgery + RT + CTX resulted in survival rates of 47.8% (Figure 3).

### 4.3 Prognostic Factors in PTC Variants Patients

**FIGURE 4:**
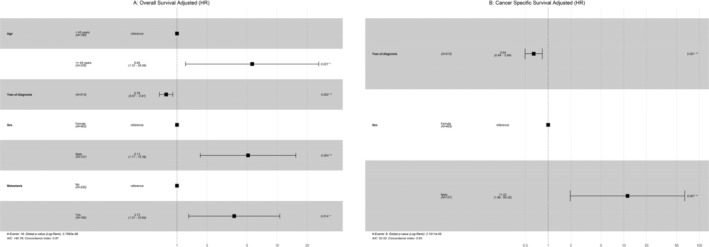
HCC: (A) Multivariate Cox regression for overall survival; (B) Multivariate Cox regression for cancer‐specific survival (CSS).

**FIGURE 5:**
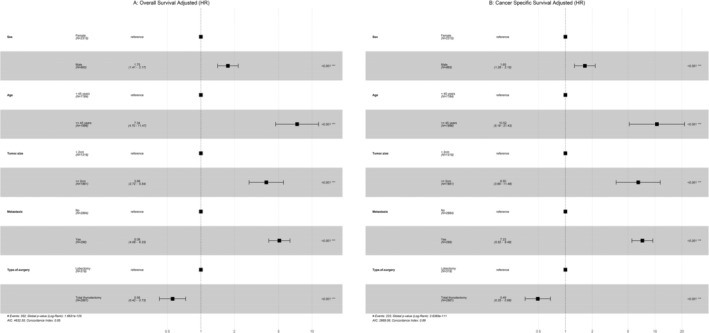
CCV: (A) Multivariate Cox regression for overall survival; (B) Multivariate Cox regression for cancer‐specific survival (CSS).

To identify potential independent predictors of PTC variants, we used a univariate Cox regression model to identify significant factors and used them in the multivariate Cox regression. Older age, male sex, and metastasis were poor prognostic factors for OS and male sex for CSS in HCC patients (Figure 4). In terms of CCV, older age, male sex, large tumor size, and metastasis were poor prognostic factors for OS, whereas total thyroidectomy was a good prognostic factor for OS. N1, unmarried status, surgery + RT + CTX, Metastasis, and total thyroidectomy were poor prognostic factors for CSS, whereas surgery + RT was a good prognostic factor for CSS (Figure 5).

### 4.4 Model Performances and Interpretability

**FIGURE 6:**
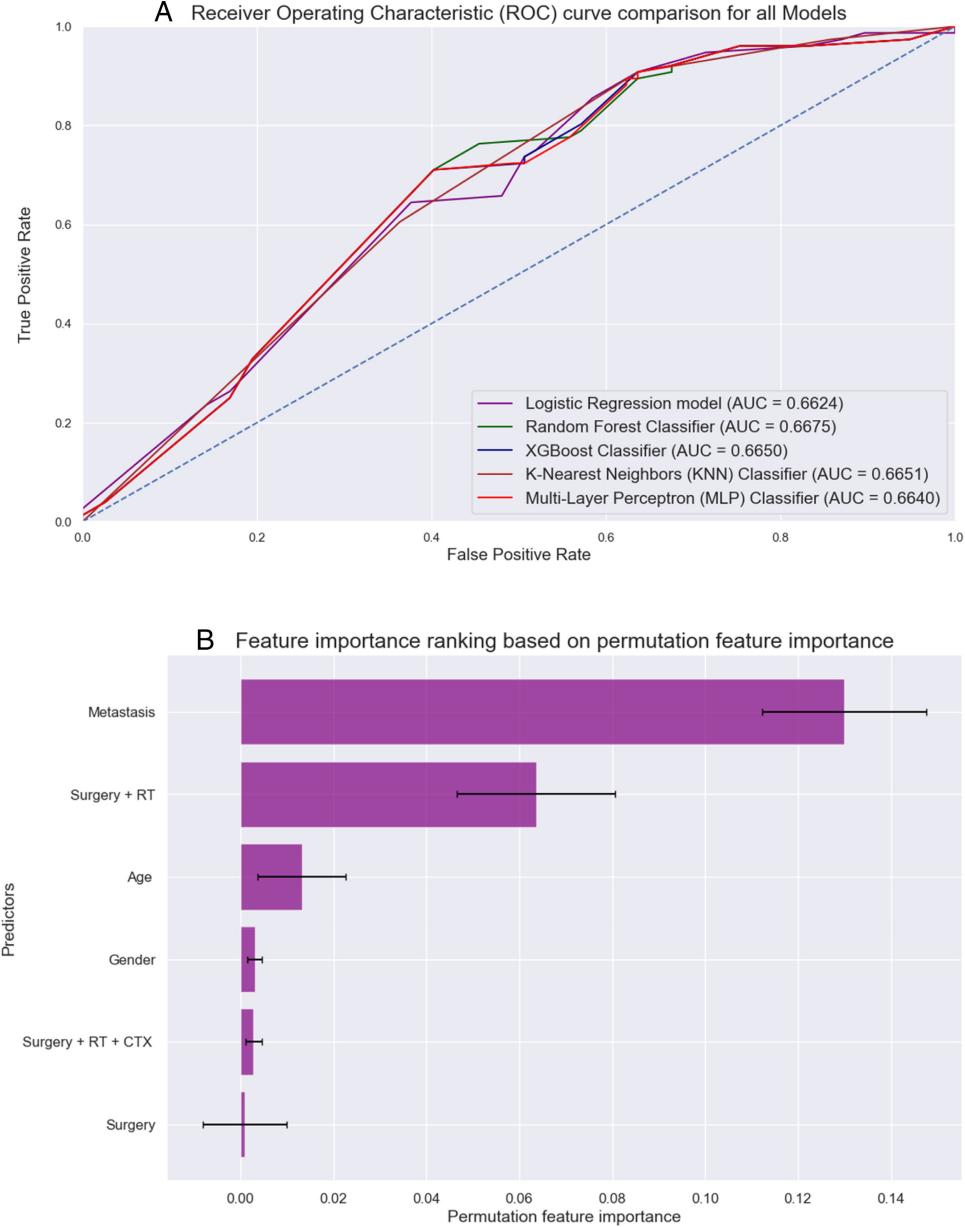
HCC: (A) Receiver operating characteristic (ROC) curves of all machine learning models (MLMs); (B) Permutation features importance (random forest classifier).

**FIGURE 7:**
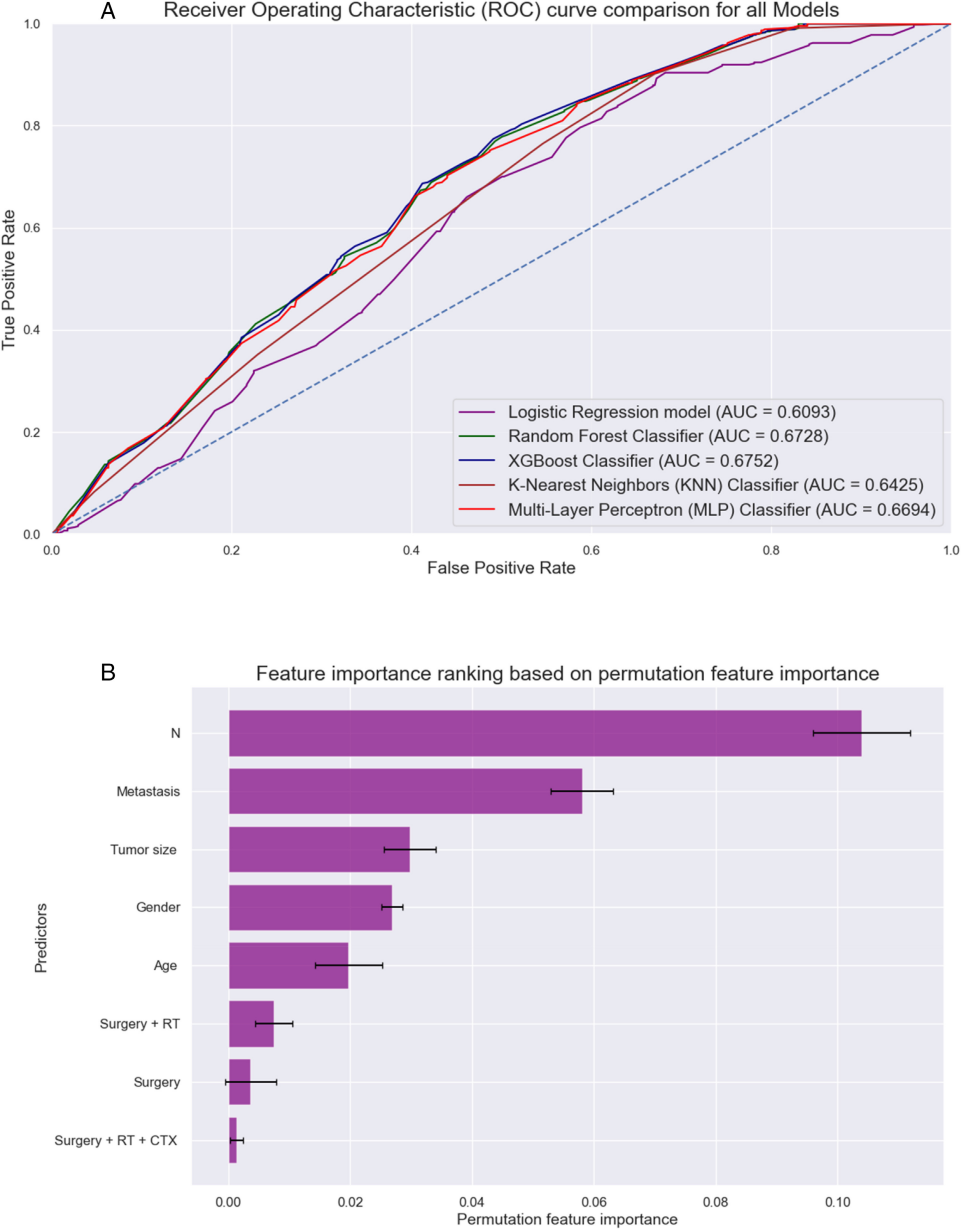
CCV: (A) Receiver operating characteristic (ROC) curves of all machine learning models (MLMs); (B) Permutation features importance (random forest classifier).

Detailed performance metrics for all ML algorithms are summarized in Table 2. ROC curves of all MLMs are displayed in Figures 6A and 7A. The most contributing features in predicting seizures in the RFC model are displayed in Figures 6B and 7B. The most significant factors for HCC were the metastasis, surgery + RT, and age, whereas for CCV, N component of TNM, metastasis, and tumor size. A web‐based tool was developed using a random forest model that incorporates various clinical variables to predict the survival of patients diagnosed with PTC variants. Clinicians and researchers can input patient‐specific data to estimate the survival duration. The interactive tool, designed for ease of use and rapid calculation, is accessible through the following link: https://sakhrshwayyat.shinyapps.io/Papillary_Thyroid_Cancer_Variants/.

**TABLE 2:** Machine learning algorithms performance.

Histology type	Model	Accuracy	Precision	Recall	F1 score	AUC
HCC	Logistic regression	67.65%	62.50%	74.47%	67.96%	0.7097
	Random forest classifier	81.37%	78.00%	82.98%	80.41%	0.8832
	XGBoost classifier	72.55%	63.38%	95.74%	76.27%	0.7747
	K‐nearest neighbors (KNN)	75.49%	71.15%	78.72%	74.75%	0.7841
	Multi‐layer perceptron (MLP)	72.55%	67.27%	78.72%	72.55%	0.7708
CCV	Logistic regression	67.14%	64.31%	73.23%	68.48%	0.7277
	Random forest classifier	82.70%	80.67%	84.84%	82.70%	0.9073
	XGBoost classifier	82.23%	74.20%	97.42%	84.24%	0.8867
	K‐nearest neighbors (KNN)	76.42%	72.60%	82.90%	77.41%	0.8244
	Multi‐layer perceptron (MLP)	77.20%	74.63%	80.65%	77.52%	0.8593

## 5 Discussion

PTC along with its various histological subtypes represents the most common subtype of thyroid cancers. The landscape of PTC expands beyond the classical variant, including diverse histological subtypes with distinct clinical characteristics and appropriate management strategies. Among PTC variants, HCC and CCV are considered rare variants. They stand out for their unique clinicopathological features and therapeutic considerations, particularly CCV which is considered one of the most aggressive variants. This study explores the efficacy of various treatment options for these PTC variants and the possible prognostic factors, shedding light on crucial aspects for clinical decision‐making and paving the way for future research endeavors [4, 17, 18].

The demographic distribution of the study population is essential for understanding the epidemiology of PTC concerning HCC and CCV. With a median age of 52 years, the prevalence of these carcinomas is notably higher in the older age group. This aligns with the general trend of increasing thyroid cancer incidence with advancing age, as observed in various populations, and is backed by a meta‐analysis that found the mean age of people with HCC was 57.6 years old at diagnosis [19]. Also, another article by Coca‐Pelaz et al. revealed that CCV commonly occurs in the 5th‐6th decade of life, further supporting our finding [20]. This may be linked to cellular aging, cumulative exposure to risk factors such as radiation, or even increased detection due to increased awareness or technological advancements in terms of screening. It was also noted that both HCC and CCV are more common among females [21, 22, 23]. This may be explained by hormonal factors, genetic differences, or increased medical surveillance among women.

The reported 5‐year survival rates provide valuable insights into the prognosis of CCV and HCC. The exceptionally high 5‐year survival rate of 97.5% for HCC patients is consistent with the typically favorable outcome of HCC compared to other PTC subtypes, as opposed to CCV where the 5‐year survival rate was lower (90.6%) [24, 25]. However, there was a variation in the survival rates when categorized according to treatment approach. The variation was more significant in the CCV subtype where surgery combined with RT yielded the highest survival rate, while surgery with RT and CTX yielded the lowest survival rate. This striking discrepancy highlights the need for further investigation into the role of chemotherapy in CCV, as the current literature lacks consensus on its efficacy. Yet, a study concluded that using chemotherapy for HCC has been disappointing, meaning that this may be the case for CCV as well [26]. Additionally, specific surgical interventions demonstrated significantly distinct survival outcomes, with lobectomy achieving a success rate of 92.5%, and total thyroidectomy displaying a higher survival rate (97.5%). A systematic review determined that total thyroidectomy of PTC reduces the chance of tumor recurrence [27]. Moreover, another study stated that lobectomy is sufficient for small unifocal intrathyroidal tumors, otherwise, total thyroidectomy is the better option [20]. Additionally, one study mentioned that total thyroidectomy would be more appropriate for the aggressive variants of PTC as CCV [28].

On the other hand, in HCC, the overall 5‐year survival rates remained remarkably high, with surgery alone and the combination of surgery and RT exceeding 96%. One study suggested that adjuvant RT may be considered for patients with high‐risk features, such as vascular invasion or distant metastasis [29]. This idea was also supported by other studies that mentioned that RT has shown improvements in survival, particularly in patients with distant metastasis [30, 31, 32]. One study discovered that radioactive iodine therapy does provide a survival benefit, but external beam radiation does not [33]. Consequently, this infers that the type of radiotherapy matters.

The nearly equal 5‐year survival rates associated with lobectomy and total thyroidectomy in HCC cases (46.9% and 48.6%, respectively) raise questions about the optimal surgical approach in these cases. This was also the case in a study done by Zhou et al., but other studies found that a total thyroidectomy is superior to a lobectomy particularly when the HCC is no longer minimally invasive, and because it has been shown that total thyroidectomy is associated with a lower recurrence rate [6, 22, 26, 34, 35]. In a study by Oluic et al., it was revealed that performing a total thyroidectomy as a primary treatment approach is actually a favorable prognostic factor [24]. However, according to our study, the results suggest that the specific treatment choice for HCC may hold less weight compared to CCV.

Identifying prognostic factors is important for tailoring treatment strategies and predicting outcomes in PTC. Male sex, older age, and metastasis were consistently associated with decreased survival in both CCV and HCC. These findings align with established risk factors for poor prognosis in thyroid cancer [6, 21, 22, 36, 37, 38, 39]. Older age may be a poor prognostic factor since older patients are more likely to have other medical conditions that can affect their survival. Additionally, older patients may be less tolerant of the stress of surgical interventions and the side effects of radiotherapy or chemotherapy.

Based on the regression models, regarding prognostic factors for HCC patients specifically, the presence of N1 had a negative impact on survival, which was supported by other studies as well [22, 33, 35]. Nodal involvement can be thought of as a stepping stone for the cancer to spread further and potentially lead to metastasis, thus affecting the survival rate.

On the other hand, the regression models for CCV cases revealed that large tumor size was associated with poor prognosis. The association of the black race with poor prognosis in thyroid cancer has been reported in previous studies [36]. However, our study did not identify race as a significant prognostic factor. Moreover, CCV was associated with a favorable prognosis for patients who underwent surgery plus radiotherapy or total thyroidectomy. Furthermore, ML can be utilized as a transformative tool in various aspects of the medical field. It has been used in other studies either for classifying thyroid cancers or to find factors that impact PTC recurrence [40, 41]. In our study, various factors were integrated into different ML models to determine which factors influence survival the most in CCV and HCC. Interestingly, metastasis status was the most important one in both variants and this is supported by other studies that considered advanced staging a prognostic factor according to multivariate Cox regression or nomogram models [6, 39].

Despite the comprehensive nature of this study, several limitations should be acknowledged. The retrospective nature of the analysis introduced inherent biases and limitations associated with data extraction from the SEER database. Additionally, the doses of RT and CTX were not included; therefore, we were unable to determine whether certain doses were better for a certain variant. However, this study had several strengths. First, it addresses two rare and aggressive PTC variants, offering valuable knowledge for their management and prognosis. Second, consideration of multiple clinicopathological factors and treatment approaches allows for a deeper understanding of their relationship with survival outcomes. Third, integrating ML enhances the analysis by predicting survival and identifying the key factors affecting it. Finally, the study's large dataset from SEER strengthens its statistical power and generalizability.

## 6 Conclusion

Our study of PTC provides significant insights into its variants. HCC shows high survival rates, suggesting that the treatment type has less impact, whereas CCV presents varied outcomes, emphasizing the need for tailored treatments. This underscores the uncertainty surrounding the efficacy of chemotherapy in CCV management and highlights the need for further research. Key prognostic markers such as age, sex, tumor size, and metastasis shape these outcomes and are crucial for personalized treatment strategies. The integration of ML into clinical practice is a critical tool for predicting outcomes and identifying prognostic factors, ultimately improving patient outcomes for PTC variants.

## Author Contributions

**Sakhr Alshwayyat:** conceptualization, formal analysis, methodology, visualization, writing – original draft. **Haya Kamal:** writing – original draft. **Owais Ghammaz:** formal analysis, software. **Tala Abdulsalam Alshwayyat:** writing – original draft. **Mustafa Alshwayyat:** formal analysis, software. **Ramez M. Odat:** writing – review and editing. **Hamdah Hanifa:** writing – review and editing. **Wafa Asha:** supervision, writing – review and editing. **Nesreen A. Saadeh:** supervision, writing – review and editing. All authors have read and agreed to the published version of the manuscript.

## Ethics Statement

The ethical approval and informed consent statements are not applicable for this type of research. Authorization and data were obtained through the SEER website and database, respectively.

## Conflicts of Interest

The authors declare no conflicts of interest.

## Data Availability

The data that support the findings of this study are available from the corresponding author upon reasonable request.
